# Kentucky Medical Society

**Published:** 1852-10

**Authors:** 


### KENTUCKY MEDICAL SOCIETY.

OUR Kentucky brethren, we trust, will not forget the meeting of the State Medical Society, which is to take place in this city on the 20th instant. We owe it to ourselves to foster and sustain this society. In several States younger than Kentucky, similar societies are in successful operation, conferring benefits upon the profession, and honor upon their members. We predict that the papers and reports read at the approaching meeting, will be of a character to reflect credit upon the profession in Kentucky. Association, harmony, and concert of action, this is the secret of success. We shall advance medicine in proportion as we unite our efforts to the common end—as we meet together and communicate knowledge, correct errors, start inquiries, encourage investigation, reward discoveries, and mutually fire and stimulate the minds of one another. The London Philosophical Society, the British Association, the Institute of France—how much does the world owe to these societies; and the American Associations, Medical and Scientific, young as they are, how much have they already done to advance the science of our country! Let every physician feel that he has an interest in, and owes a duty to the Medical Society of his State.

